# ZikaPLAN: Zika Preparedness Latin American Network

**DOI:** 10.1080/16549716.2017.1398485

**Published:** 2017-12-13

**Authors:** A. Wilder-Smith, R. Preet, K. E. Renhorn, R. A. Ximenes, L. C. Rodrigues, T. Solomon, J. Neyts, L. Lambrechts, H. J. Willison, R. Peeling, A. K. Falconar, A. R. Precioso, J. Logan, T. Lang, H. P. Endtz, E. Massad

**Affiliations:** a Unit of Epidemiology and Global Health, Department of Public Health and Clinical Medicine, Umeå University, Umeå Sweden; b Instituto de Apoio a Fundacao, Universidade de Pernambuco, Recife, Brazil; c London School of Hygiene and Tropical Medicine, London, UK; d Institute of Infection and Global Health, The University of Liverpool, Liverpool, UK; e Rega Institute for Medical Research, Department of Microbiology & Immunology, Katholieke Universiteit Leuven, Leuven, Belgium; f Institut Pasteur, Insect-Virus Interactions Group, Department of Genomes and Genetics, CNRS Unité de Recherche Associée 3012, Paris Cedex 15, France; g Institute of Infection, Immunity & Inflammation, University of Glasgow, Glasgow, UK; h Departmento del Medicina, Fundacion Universidad del Norte, Barranquilla, Colombia; i Instituto Butantan, Brazil; j The Global Health Network, Masters and Scholars of the University of Oxford, Oxford, UK; k Fondation Mérieux, Lyon, France; l Department of Medical Microbiology & Infectious Diseases, Rotterdam, The Netherlands; m Fundacao de Apoio a Universidade de Sao Paulo, Sao Paulo, Brazil

**Keywords:** Zika, congenital Zika syndrome, public health emergency, epidemic preparedness, research capacity building network, collaboration, European Commission

## Abstract

The ongoing Zika virus (ZIKV) outbreak in Latin America, the Caribbean, and the Pacific Islands has underlined the need for a coordinated research network across the whole region that can respond rapidly to address the current knowledge gaps in Zika and enhance research preparedness beyond Zika. The European Union under its Horizon 2020 Research and Innovation Programme awarded three research consortia to respond to this need. Here we present the ZikaPLAN (Zika Preparedness Latin American Network) consortium. ZikaPLAN combines the strengths of 25 partners in Latin America, North America, Africa, Asia, and various centers in Europe. We will conduct clinical studies to estimate the risk and further define the full spectrum and risk factors of congenital Zika virus syndrome (including neurodevelopmental milestones in the first 3 years of life), delineate neurological complications associated with ZIKV due to direct neuroinvasion and immune-mediated responses in older children and adults, and strengthen surveillance for birth defects and Guillain–Barré Syndrome. Laboratory-based research to unravel neurotropism and investigate the role of sexual transmission, determinants of severe disease, and viral fitness will underpin the clinical studies. Social messaging and engagement with affected communities, as well as development of wearable repellent technologies against *Aedes* mosquitoes will enhance the impact. Burden of disease studies, data-driven vector control, and vaccine modeling as well as risk assessments on geographic spread of ZIKV will form the foundation for evidence-informed policies. While addressing the research gaps around ZIKV, we will engage in capacity building in laboratory and clinical research, collaborate with existing and new networks to share knowledge, and work with international organizations to tackle regulatory and other bottlenecks and refine research priorities. In this way, we can leverage the ZIKV response toward building a long-term emerging infectious diseases response capacity in the region to address future challenges.

## Background

The 21st century has seen an unparalleled number of emerging infections – the latest is the Zika virus (ZIKV) epidemic in Latin America and South Pacific Islands [1]. The unusual neurotropism of this flavivirus resulting in clusters of neurodevelopmental birth defects, further compounded by clusters of severe neurological disease in adults, triggered the World Health Organization (WHO) to declare a public health emergency of international concern (PHEIC) in February 2016. The ongoing ZIKV outbreak has exposed the challenges associated with the implementation of urgently needed research in the Latin American region, and has underlined the need for a coordinated research network across the whole region that can rapidly respond to emerging threats. Such a network would not only facilitate research to investigate ZIKV but also be available to respond rapidly to any future emerging threats in Latin America.

For this reason, in January 2016 the European Commission announced a funding call, with the specification to set up a research network across the Latin America region to facilitate, coordinate and implement urgent research against the current ZIKV outbreak, and eventually lay the foundation for a preparedness research network against any future emerging severe infectious threats. The scope of the call can be found in Box 1: Call Topic SC1-PM-22-2016. The submission deadline was 28 April 2016; on 21 October 2016 the European Commission formally announced that three consortia were awarded: ZikaPLAN (www.zikaplan.tghn.org), ZIKAction (www.zikaction.org), and ZIKAlliance (www.zikalliance.tghn.org). Here we present ZikaPLAN, the composition of the consortium and its objectives and research design.

**Figure 1.**
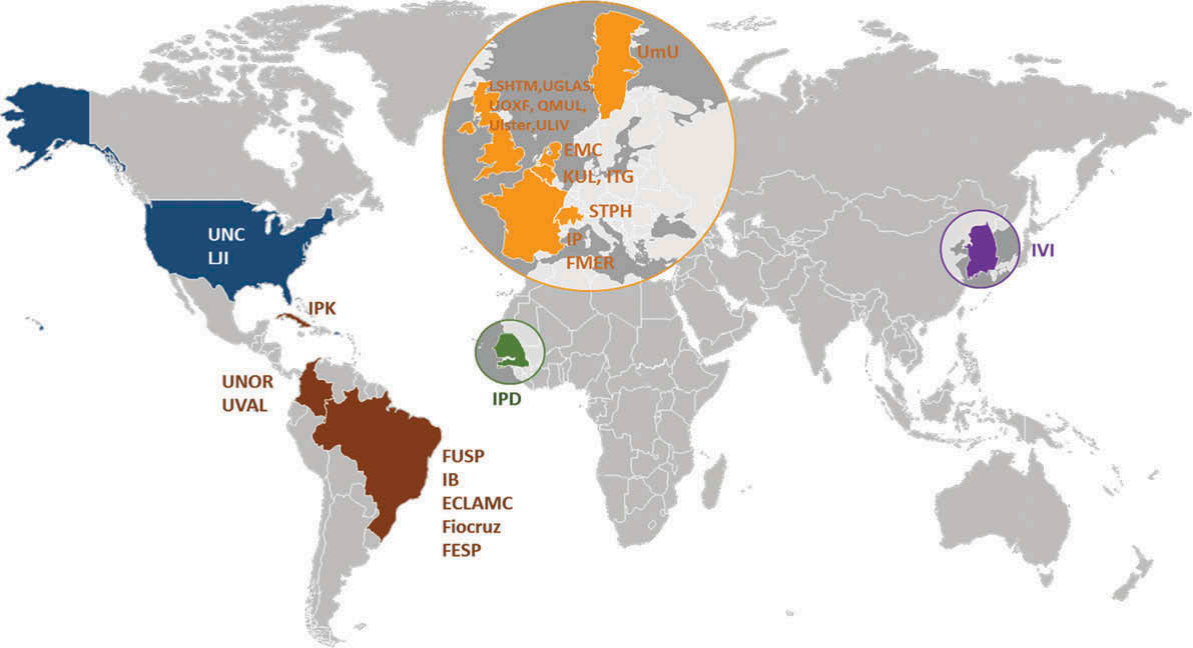
Geographic distribution of the consortium.

**Figure 2.**
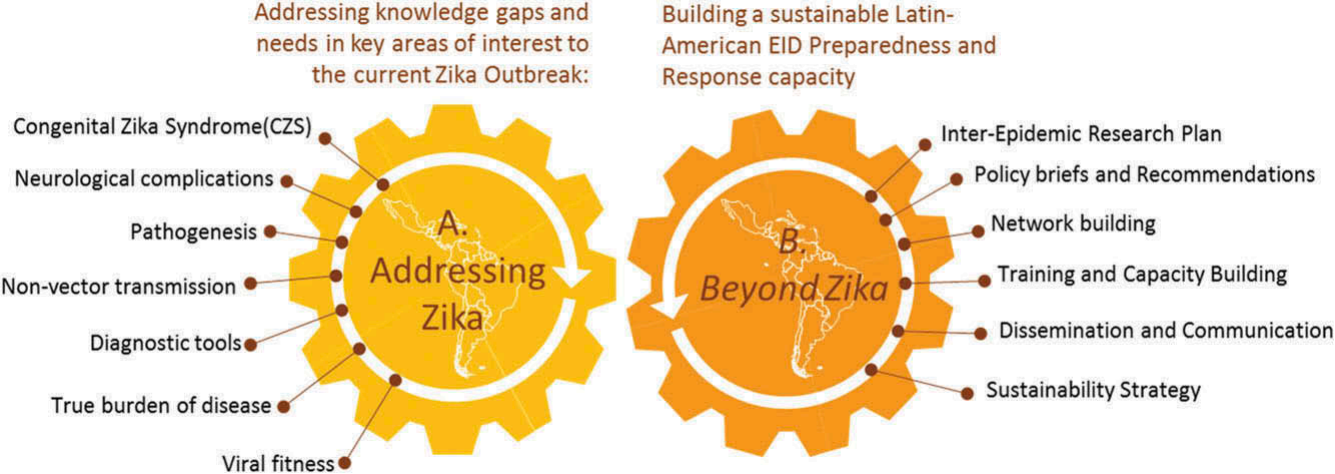
Dual purpose of ZikaPLAN.

The ZikaPLAN initiative combines a multinational and interdisciplinary consortium consisting of 25 institutional partners in Latin America, North America, Africa, Asia, and various academic centers in Europe. The institutions are listed in Box 2, and the geographic distribution of the consortium is shown in Figure 1. The hosting institution is Umeå University, Sweden and the Scientific Coordinator and Scientific Co-coordinator are Professors  Annelies Wilder-Smith and Eduardo Massad respectively with Dr Raman Kaur Preet as the Executive Director. The project, the consortium, and its main components have been designed with these dual, complementary purposes in mind (Figure 2).

The consortium consists of experts with a broad range of expertise covering virology, entomology, arboviral diseases, clinical trial expertise, birth defect surveillance, clinical cohort studies, pediatrics, neurology, qualitative research, and communications. Many of these experts already collaborated in a previous European Union (EU)-funded consortium called DengueTools [2], and many experts in this consortium serve as advisors to the WHO related to Zika. Even prior to the EU research funding, many consortium members had already conducted Zika related work and published extensively on Zika (check under ‘publications’ at: https://zikaplan.tghn.org).

(A) Addressing ZIKA: we will address the current knowledge gaps in ZIKV as requested in the call topic: association with congenital syndromes and neurological complications, clinical spectrum of disease, determinants of severe disease, pathogenesis, and in particular neuro-pathogenesis, nonvector and vector transmission, diagnostics innovation and evaluation, burden of disease, and risk factors for geographic spread; birth defect surveillance, social science for communication strategies with affected communities, novel personal preventive measures, and modeling on vector control and vaccine strategies for evidence-informed policies.

(B) Preparing for beyond Zika: While addressing Zika, we will position the various activities and results as a springboard toward building a sustainable Latin American network capable of rapidly launching concerted, large-scale research responses to emerging infectious diseases outbreaks of significant (potential) impact to the region. This way, we can leverage the Zika response toward building a long-term emerging infectious diseases (EID) response capacity in the region beyond the 4-year project period.

## Concept and methodology

**Figure 3.**
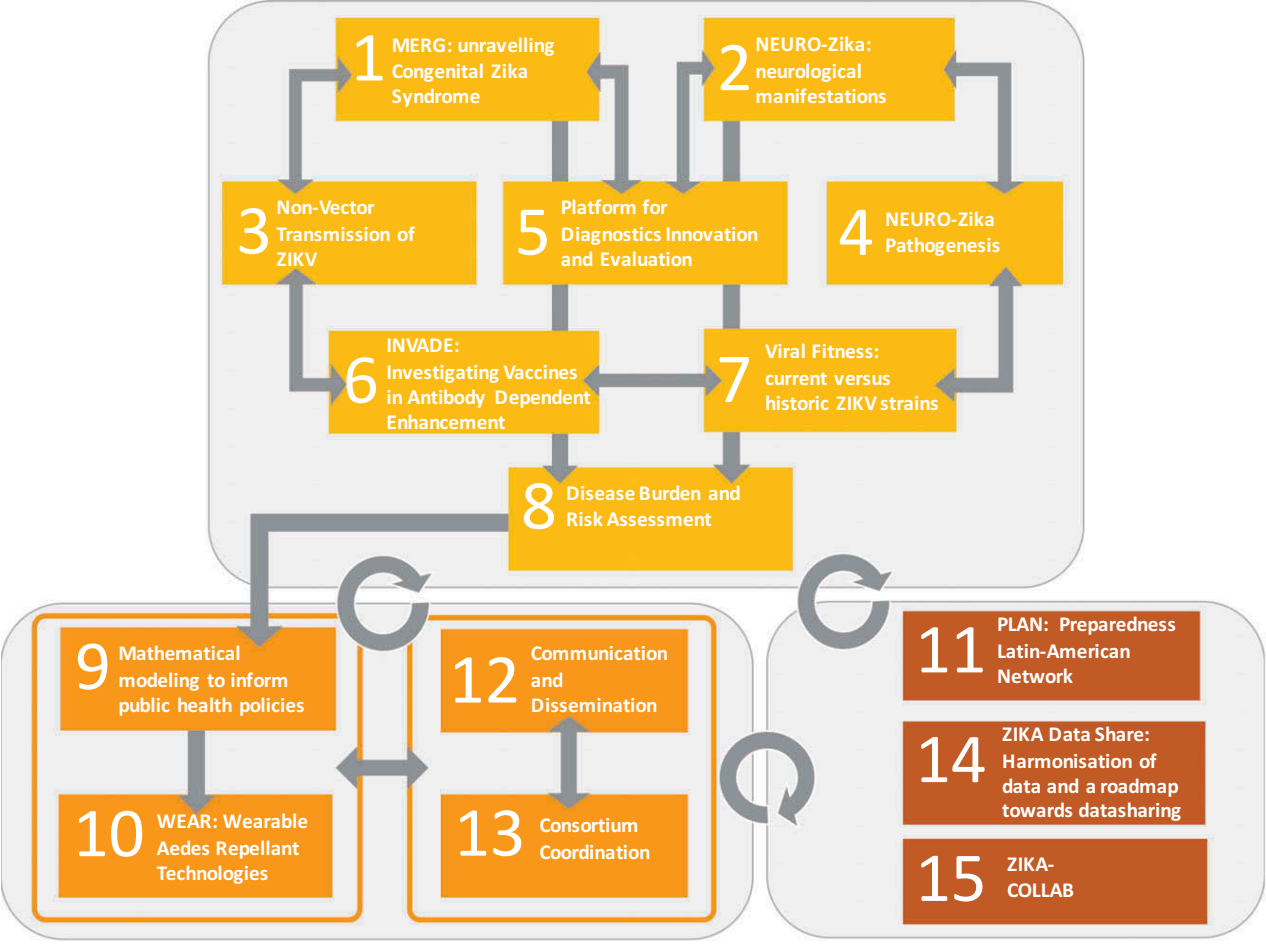
Schematic work plan by WP.

The project’s work plan is built on 15 interoperating work packages (WP). The structural design of ZikaPLAN reflects the ambition to address the urgent research gaps (WP 1–8), identify short- and long-term solutions (WP 9–10), and build a single sustainable Latin American EID Preparedness and Response capacity, WP 11. Communications and consortium coordinated are handled in WP 12 and 13 respectively. The project collaborates and interlinks with the two other EU-funded Zika consortia, ZIKAction and ZIKAlliance on protocol development, data sharing, joint data-analysis plans, and biobanking, and aims to develop commonalities for the dissemination of research findings (WP 14–15). Figure 3 presents the schematic work plan by WP. Box 3 summarizes the primary research objectives of WP 1–12.

## Description of WP

### WP 1 Microcephaly Epidemic Research Group (MERG): unraveling congenital Zika syndrome

WP 1 aims to address the main concern of ZIKV infections that led to the declaration of a PHEIC: adverse fetal outcomes. This work package is conducted by the Brazilian MERG [3], which was established in response to the first alert of microcephaly in Recife/North-East Brazil in 2015 (University of Pernambuco, London School of Hygiene and Tropical Medicine), and is devoted to the clinical recognition of congenital Zika syndrome. MERG recruits pregnant women presenting with rash and laboratory-confirmed ZIKV infection in a number of sites in Brazil. Its objectives are to determine the absolute risk of miscarriage, still birth, microcephaly, and other clinical manifestations of adverse outcomes to fetuses born to pregnant women with ZIKV infection by gestational age; and to define the full spectrum of congenital Zika syndrome at birth and the subsequent manifestations and neurodevelopmental milestones in the first 3 years of life [4–14]. This will help develop an improved case definition of congenital Zika syndrome for surveillance purposes and birth-defect registries, and help us understand the extent of disability and the social and economic burden to affected families.

### WP 2 neuro-Zika: neurological manifestations of Zika

The ‘Neuroviruses Emerging in the Americas Study’ (NEAS) is a multicentre study in the Americas (NCT03206541) to establish a comprehensive registry of the clinical, radiologic, and paraclinical profile of patients with new onset of neurological diseases associated with Zika, dengue and chikungunya viruses (http://www.neasstudy.org/en/home/) with a network involving more than 10 hospitals in Colombia. NEAS was initiated by Prof. Carlo Pardo-Villamizar from Johns Hopkins University, US, and has already resulted in various publications [15–17]. The NEAS network will be enhanced by various additional sites in Brazil. The main objective is to define the full spectrum of neurological complications due to ZIKV by using a case-control study design. This potential spectrum includes immunologically mediated illness, for example Guillain–Barré syndrome (GBS) or acute disseminated encephalomyelitis (ADEM), and direct viral invasion of the nervous system for example meningoencephalitis and neuropathies. The International GBS outcome study (IGOS) https://gbsstudies.erasmusmc.nl/, based at Erasmus University, the Netherlands, will adapt some of its protocols to the Latin American context and make them widely available to neurologists and expand the network to increase the number of participating hospitals so that improved case-management protocols, larger sample sizes for biomarker studies, and a coordinated and harmonized effort for well-designed studies will be achieved that will benefit Latin America beyond the immediate response to ZIKV. Several investigators in this work package are members of the WHO Guideline Development Group for GBS and other neurological diseases associated with ZIKV, and are experts in the clinical care and management of patients with GBS and other neurological sequelae of central nervous systems (CNS) viral infection. The implementation of the research in this work package will strengthen the management of these conditions across Brazil and Colombia, working in conjunction with the WHO Expert Panel for ZIKV infections. For example, clinical guidelines for the harmonization of management of patients with Neuro-Zika across Latin America will be produced.

### WP 3 nonvector transmission in humans and mice models

The incidence rate of ZIKV semen infection in male returning travelers with laboratory confirmed ZIKV infection will be determined as a proportion of the total number of included men with confirmed ZIKV infection. The replication fitness of ZIKV in semen will be explored by isolation of ZIKV virions in culture. The persistence of ZIKV in semen after acute infection will be analyzed semiquantitatively, and the incidence of secondary cases reported. Furthermore, this WP will establish a ZIKV mouse model for sexual transmission and study the pathway of entry of the virus via the vaginal route. Furthermore, a vertical transmission mice model will be developed to study whether an infected fetus contributes to higher viremia in the mother.

### WP 4 neuropathogenesis

WP 4 will investigate pathomechanisms of neurotropism in the laboratory through a combination of approaches in virology, animal studies, neuroimmunology, cellular neuroscience, and host–virus interaction studies including neural receptors. Through close integration and specimen sampling through WP2 enhanced by mice models, the specific objectives are: (1) to define Zika virus tropism in the peripheral (PNS) and CNS in vitro and in vivo; (2) to determine the consequences for PNS and CNS cell health, function and survival of direct ZIKV infection, in vitro and in vivo; (3) to investigate intra- and intercellular trafficking of ZIKV within and between the periphery and the PNS and CNS; (4) to characterize the nature of ZIKV’s main cellular receptors on human neural cells, screening proteins, glycoproteins, proteoglycans, glycolipids, and thereby identify candidates used by ZIKV to attach to neural cells; (5) to determine if humoral factors from Zika-GBS patient and control sera are pathogenic to PNS and CNS cells in vitro and ex vivo; (6) to examine sera from Zika-GBS cases and controls for antigen targets known to be associated with conventional GBS, principally peripheral nerve glycolipids, using sensitive immunoassays; and lastly (7) to examine differential immune responses in patients with nonneurological ZIKV infection, patients with viral invasion of the CNS (e.g. encephalitis), and patients with autoimmune neurological disease. Understanding the intricate relationships between direct neuroinfection and postinfection-related neural autoimmunity, and exploring the scientific principles that underpin the clinical phenotypes associated with this dichotomization will have a profound impact on developing approaches to diagnosis, treatment, and vaccine development. We hope to identify relevant biomarkers, and possibly neural receptors – which could be potentially exploited for prognostic use (biomarkers) or therapeutic intervention (targeting neural receptors).

### WP 5 platform for diagnostics innovation and evaluation

The platform for diagnostics innovation and evaluation is a major focus of ZikaPLAN. Regarding diagnostics innovation, we aim to identify the major type-specific and cross reactive B-cell epitopes on ZIKV for development of minimally cross-reactive IgM and IgG for more specific serological assays. We also hope to identify monoclonal antibodies to NS1 antigen for the potential development of a NS1 based assay – all with the aim to develop novel ZIKV diagnostic tests in accordance with WHO Target Product Profiles.

Our evaluation platform will be based upon well-characterized archived specimens obtained by informed consent, in accordance with the protocols developed by the WHO prequalification program for this purpose. All the evaluation sites will be compliant with the principles and practice of Good Clinical Laboratory Practice (GCLP). Assays that fulfill the target performance set by WHO will be selected to proceed to clinical performance studies, which will be conducted at our virtual network laboratories that include the Pan American Laboratory Network for Dengue and other arboviruses, RELDA (which comprises 18 laboratories in Latin America and five in the Caribbean), the WHO collaborating center for arboviral diseases in Senegal (IPD), ITG Antwerp, and the International Vaccine Institute Korea (IVI) – all with access to a geographic diversity of samples to test for crossreactivity against an array of diverse flaviviruses. Samples from these sites will form a virtual biobank with a strict code of governance on the use of samples to facilitate test development, evaluation, and research. A manual and standard operating procedures on sample collection and characterization will be developed to ensure harmonization of bio-banking procedures. This platform will be used for evaluation of commercially developed new diagnostic assays (at a fee) and will be free to the wider academic community under certain contractual conditions. The evaluation results will be provided to WHO for prequalification and regulatory authorities in countries for approval.

### WP 6 INVADE: investigating vaccines in antibody-dependent enhancement

‘INVADE’ sets out to carry out T-cell and B-cell epitope mapping, and assess the role of flavivirus antibodies in protective and pathogenic immunity and the role of antibody-dependent enhancement (ADE) [18]. In particular, the aim is to define the epitope breadth/repertoire and the targets of CD4 and CD8 responses; to derive sets of HLA class I and class II predicted epitopes covering the entire ZIKV proteome; to define phenotypes and T-cell subsets associated with CD4 and CD8 specific T cells; to identify the major type-specific and crossreactive B-cell epitopes on ZIKV, and to assess the role of flavivirus antibodies in protective and pathogenic immunity (e.g. related to prior dengue infections, dengue, and yellow fever vaccines). More than 2000 T-cell epitopes [3,4] and epitopes targeted by type-specific neutralizing, nonneutralizing, and possibly enhancing/autoimmune reactive antibodies will be identified, which may help develop vaccines and study potential mechanisms of immune pathogenesis responsible for severe neurological and fetal defects.

### WP 7 viral fitness: current versus historic ZIKV

The aim of WP 7 is to investigate whether ZIKV emergence is associated with adaptive viral evolution in the vector, i.e. viral fitness differences that may explain the observed recent increase in ZIKV transmission by mosquitoes among humans. We will also address whether contemporary ZIKV result in more severe neurological disease, different tissue tropism, and higher viral loads in mice compared with historic ZIKV isolates.

### WP 8 ‘disease burden and risk assessment’

This WP assembles a wealth of data obtained from various sources, including a longitudinal cohort study of 17,000 subjects aged 2–59 years in 14 different geographic locations in Brazil over 3 years, recruited through an ongoing Phase 3 trial by ZikaPLAN Partner Butantan. Seroprevalence studies will help to assess the asymptomatic to symptomatic ratio, the age- and gender-stratified attack rates, the clinical spectrum, and the impact of preceding dengue infections on the severity of illness. Documentation of the evolving epidemiology of ZIKV will be enriched by data from the Latin American network on birth-defect surveillance (ECLAMC). We will establish an ‘International Committee for Birth Defect Surveillance Tools for Infection Disease Response Preparedness’. This will consist of representatives from four birth-defect surveillance networks with expertise in this area: The European network of population-based congenital anomaly registries (EUROCAT), ECLAMC (Latin America), both of which are ZikaPLAN partners, and the International Clearinghouse for Birth Defects (ICBDSR), and Centre for Disease Control (US). A detailed inventory of Birth Defect Surveillance Tools will be made available on The Global Health Network (TGHN) web platform. A new Tablet App will be produced, tested, and used for the diagnosis and coding of birth defects in low-resource settings with good access to pediatricians. The App will contain atlas/picture material, diagrams created digitally by a medical illustrator, the International Classification of Disease Codes version 10, training videos, and guides. A specific ‘Zika module’ will also be created. The App will be an important part of the sustainability in terms of globally available tools for infectious disease preparedness and the surveillance of birth defects in low-resource settings.

The Swiss Tropical and Public Health Institute (Swiss TPH) will establish a smartphone-based sentinel surveillance system in travelers to Latin America and other Zika-affected countries in order to assess the incidence of Zika-/arboviral-related symptoms in travelers, trace and diagnose these travelers, and assess the risk of zika/arbovirus introduction to a vector-infested region of Europe (Barcelona, Spain). Swiss TPH will also develop baited filter-paper-based mosquito traps for the collection of mosquito saliva in tropical and subtropical countries (using international travelers), in order to assess the arbovirome of the local mosquito populations.

The results from our partners and this work package, further enhanced by literature reviews, will feed into mathematical modeling to document and predict burden of disease in Latin America. Complemented by data from GeoSentinel, Healthmap, and Bluedot, we will analyze the geographic spread of Zika infections beyond Latin America. Much of this will involve modeling spread via air passengers [19,20]. We will apply previously developed models for the risk of dengue spread via air passengers [21–23] to model the spread of ZIKV. We already modeled the risk of ZIKV infection during the Olympics [24,25] and provided input to the WHO IHR committee not to cancel the Olympics. We also published the most likely time of ZIKV introduction into Brazil based on mathematical modeling [26].

### WP 9 mathematical modeling to inform public health policies

Here, we will model best vector control strategies for high-population-density areas in Latin America. To this end, work on vectorial capacity of *Aedes* mosquitoes for Zika in temperate countries has already been conducted by the ZikaPLAN team [27]. Modeling ecological, climate, and mobility patterns will assist in enhancing appropriate public health responses [27–29]. We will model vector-control strategies (Wolbachia/genetically modified mosquitoes) to inform optimal control bundles to mitigate future outbreaks, in particular in Latin American cities. Furthermore, this WP aims to establish mathematical models on the role of sexual transmission in countries without *Aedes aegypti/albopictus* mosquitoes [30].

We will also build a community of practice related to vector control. This community of practice for vector-control groups will be called The Global Vector Hub and will be located within the TGHN digital platform to maximize visibility and uptake, and also to provide users with access to further tools and resources that can support their research and teams. The community of practice for vector control is focused on supporting and linking researchers working with mosquitoes through a digital infrastructure where they can share tools and resources in order to deliver training, raise capacity, and establish standards. Enhancing vector-control strategies through such a community of practice will benefit ZIKV control but also other mosquito-borne diseases.

### WP 10 WEAR: wearable Aedes-repellant technologies

‘WEAR’ aims to develop personal protective measures for pregnant women (impregnated maternity clothing for example). We will investigate and develop novel detergents containing repellents that can be used during laundry to allow active repellents to be applied to clothing for protection and also investigate new wash-resistant technologies, including novel silica-shell, polymer fibers, and microencapsulated formulations to determine whether repellent active ingredients can be retained in fabrics for multiple washes. New plastic wearable repellent technologies will be developed and tested, including flip flops, wrist bands, and necklaces using a plastic silica technology for protection against *Aedes* spp. mosquitoes. Qualitative research will be conducted to assess the acceptability of such clothing and technologies.

### WP 11 ZIKA collaborative establishment of a Latin American preparedness network (PLAN)

‘PLAN’ led by TGHN at Oxford University will ensure that the consortium, networks built, activities performed, and results obtained are leveraged into establishing a Latin American EID Preparedness and Response network in the region beyond Zika. To this purpose, we will work together with ZIKAlliance and ZIKAction in order to leverage synergies, in particular with regard to the clinical cohort studies. A specific platform will be set up to enhance the outreach to expand to new sites and to develop into an independent research network beyond the project time. This is called REDe (the Spanish and Portuguese term for network), which will be a shared and open space for information exchange and releasing research documents such as protocols and operating procedures, to ensure standard outcome measures are used, to optimize data sharing, and also to disseminate the research tools in order that others may use and adapt them. REDe is hence a research-capacity development and knowledge-exchange space for all three EU-funded Zika consortia and their clinical sites. The bottlenecks that we will identify throughout the project are likely to be somewhat similar to those encountered in Europe, and we will therefore also liaise with EU funded Platform for European Preparedness Against (Re-)emerging Epidemics (PREPARE) to incorporate lessons learned through their preapproval processes of epidemic clinical research protocols.

Research needs to be embedded into a response to an outbreak or emerging disease [31], and to achieve this there needs to be local ability to undertake study design and operation to gather the high-quality evidence that is rapidly needed [32]. TGHN will be harnessed as a respected online platform for research education, training, and resources for capacity building in Latin America as the host of the REDe network. The aim of REDe is to link together and work with experienced and inexperienced research sites across Latin American and the Caribbean to deliver knowledge, research tools, and training. The underlying philosophy is that if there is to be a locally led and rapid research response in an outbreak, then the local ability to undertake the collection of high-quality research data needs to be built up. A wide range of cross-cutting research resources and tools will be provided, such as free online seminars and courses, downloadable training kits, articles, templates and guidance notes, in particular linkage to IGOS, ECLAMC, and diagnostics. Harmonized web-based tools for birth-defect registries will be accessible. Other tools such as ‘SiteFinder’, an application that allows partners planning studies to find new sites to collaborate with, are also freely available and easily accessible. The training center already provides translated core courses such as Good Clinical Practice, GCLP, and Bioethics, as well as broader topics such as ‘Introduction to Clinical Research’ and ‘Children and Clinical Research’. Alongside these resources and training, there will be a whole set of regionally based activities and support systems to provide opportunities for research staff from a specific region to meet, learn, and share experiences.

### WP 12 dissemination and communication

This WP will develop and implement a detailed communication and dissemination plan serving as the central consortium-wide guiding document for how we deal with dissemination and communication with our stakeholders. These stakeholders include international and national governmental organizations/health authorities/funders in the area of EID preparedness and response, e.g. WHO, PAHO, PREPARE, and Global Research Collaboration for Infection Disease Preparedness (GLoPID-R). Engagement with affected communities, in particular pregnant women, including social messaging of personal protection, will also be central to WP 12. Following qualitative research in North-East Brazil supportive, actionable messages will be developed that take data on burden of disease into account. These social messages will be based on an empirical understanding of people’s perceptions and concerns about Zika and will be built on the lessons learned from the Zika-related messages that have been used to date, in particular with those coordinated by WHO (http://www.who.int/emergencies/zika-virus/en/)

### WP 13 consortium coordination and management

Umeå University, Sweden under WP 13 will coordinate all the collaborative efforts and facilitate communication within the consortium. It will set up an effective management framework to ensure progress toward its planned objectives reflecting our contractual commitments to the European Commission.

### WP 14 Zika data share: harmonization of data and roadmap to data sharing

The aims of the work package are (1) to ensure harmonization of protocols and standardization of tools (including data dictionaries) for the multicentre pregnant women and children cohort studies across the three EU-funded Zika consortia (ZikaPLAN, ZIKAction, ZIKAlliance); (2) to conduct data sharing within the three consortia and to provide a roadmap toward wider data sharing. The pregnant women cohort is harmonized between ZIKAction and ZIKAlliance (as both recruit asymptomatic and symptomatic pregnant women), whereas ZikaPLAN recruits only women symptomatic ZIKV infection during pregnancy. The data garnered through these combined cohorts will also feed into the WHO-led ‘individual participant data meta-analysis (IPD-MA)’, which will combine de-identified, participant-level cohort data to identify and quantify the relative importance of different predictors of congenital Zika syndrome (CZS) (www.who.int/entity/reproductivehealth/projects/ZIKV.pdf). Individual participant data (IPD) allow for more consistent control of confounders and facilitate the assessment of effect measure modification and sensitivity analyses that would not have been possible if we did not combine the data obtained from the three EU-funded consortia and other consortia or research groups with similar cohort studies. Under the guidance of WHO, the use of IPD will allow us to fully explore subject-level sources of heterogeneity in the association between congenital ZIKV infection and pregnancy complications, and will facilitate the identification of the subgroups of women at the highest risk of experiencing ZIKV-related pregnancy complications.

### WP 15 ZIKA-COLLAB

The overall objective is to enhance the output of the respective EU-funded Zika consortia through shared management structures and communications activities, create and maintain joint management and oversight structures, and address ethical and information governance issues, ensuring ethical and safe care and treatment of animals and use of patient data and samples for research across and potentially outside of participating consortia. We will organize integrated communications across consortia, including the creation of a Communications Oversight Board, planning of joint meetings, and organization of cross-consortia working groups to ensure clear, coherent messages that integrate all communication and dissemination activities. Ethics is an integral part of research from beginning to end, and ethical compliance is pivotal to achieve real research excellence (http://ec.europa.eu/research/participants/data/ref/fp7/89888/ethics-for-researchers_en.pdf). Within the European regulatory framework, research ethics is based on the explicit European commitment to human rights. We therefore established a joint Ethics Advisory Board for the three EU-funded consortia, which will provide oversight and advice over ethical issues pertaining to our human and animal studies.

## Outlook

The speed in research response after the declaration of the PHEIC in 2016 was unprecedented. As the ZIKV outbreak is rapidly waning in 2017, it is even more significant that the Consortium should further capitalize on the REDe platform established in WP 11 and the experience gained through this ZIKV research response, in order to evolve into a network capable of rapidly launching a research response to future severe infectious outbreaks caused by emerging pathogens.

**Box 1.** Call topic SC1-PM-22-2016

Scope of the EU call:
The objective is to establish a multinational and multidisciplinary consortium across Latin America and other affected or at-risk regions, able to implement the urgently needed research during the ongoing ZIKV outbreak. The proposal should address all of the following issues:
The evaluation of the potentially causative *relationship between ZIKV and the severe reported complications*, as well as the exploration of the mechanisms involved or of alternative aetiologies if needed. All relevant research is possible, ranging from basic research, research in animal models, virology, and immunology studies, to a coordinated set of clinical studies (including for example prospective, cross-sectional or retrospective epidemiological or cohort studies, supported by harmonized case definitions and the development of improved ZIKV diagnosis and differential diagnosis assays, etc.) as necessary. If such an association is confirmed, the consortium should be ready to *rapidly launch additional studies* (e.g. observational studies aimed at establishing the natural history, pathogen and host determinants of severity of the disease, phase II or III interventional trials in primary and/or secondary care aimed at providing evidence for potential prevention [including vaccines] and/or treatment strategies). Depending on the evolution of the outbreak, the timeline of the proposed actions, and ZIKV research efforts implemented by other stakeholders, the proposed action plan should maintain the flexibility to address remaining research gaps against ZIKV. This should include the flexibility to include additional partners depending on the specific expertise required and/or the need to extend geographic scope. The consortium should further capitalize on the platforms established and the experience gained through this urgent ZIKV research response, in order to evolve into *a network capable of rapidly launching a research response* to future severe infectious outbreaks caused by emerging pathogens with pandemic potential or potential to cause significant damage to health and socioeconomics in the region [5]. Provisions should be made so that this initial research platform may be further developed through a comprehensive ‘interepidemic’ action plan addressing and fine-tuning the response to any obstacles identified during the ZIKV research response (e.g. resolving regulatory and other bottlenecks, development of adaptable study protocols, strengthening ICT infrastructure for communication and information exchange, developing a training programme to enhance the local partners’ capacity for laboratory and clinical research, developing a communication strategy for patient and public engagement, etc.). A comprehensive data-management framework allowing the standardized collection, storage, analysis, and sharing of data should be implemented, initially focusing on Zika data but eventually evolving as a critical part of the preparedness research network. Additionally, a sustainability strategy that would enable the continuation of the network beyond the timeline of the EU grant should be explored and developed during the project’s duration.
The consortium is expected to collaborate with relevant initiatives already-existing or under development at national, regional, and international level, in order to maximize synergy and complementarity and avoid duplication of the research efforts. If more than one proposal is successful, proposals should collaborate, and this should be indicated in the proposal.

**Box 2.** List of institutions in ZikaPLAN

Participant no.	Participant organization name	Country
1	Umeå University	Sweden
2	London School of Hygiene and Tropical Medicine	UK
3	University of Glasgow	UK
4	The Chancellor, Masters, and Scholars of the University of Oxford	UK
5	Queen Mary University of London	UK
6	University of Ulster	UK
7	Katholieke Universiteit Leuven	Belgium
8	Erasmus Universitair Medisch Centrum Rotterdam	The Netherlands
9	Institut Pasteur	France
10	Fundacion Universidad del Norte	Colombia
11	Universidad del Valle	Colombia
12	Fondation Merieux	France
13	The University of Liverpool	UK
14	La Jolla Institute for Allergy and Immunology	USA
15	The University of North Carolina at Chapel Hill	USA
16	Prins Leopold Instituut voor Tropische Geneeskunde	Belgium
17	Fundacao de Apoio a Universidade de Sao Paulo	Brazil
18	Instituto Butantan	Brazil
19	Associao Tecnica-Cientifica Estudo Collaborativo Latino Americano de Malformacoes Congenitas	Brazil
20	Fundacao Oswaldo Fiocruz	Brazil
21	Instituto Medicina Tropical Pedro Kouri	Cuba
22	Institut Pasteur de Dakar	Senegal
23	Schweizerisches Tropen- und Public Health-Institut	Switzerland
24	International Vaccine Institute	Korea
25	Fundacao Universidade de Pernambuco	Brazil

**Box 3.** Primary research objectives for WP 1–12

**Addressing ZIKA**:
Objective 1: To determine the (1) attack rate of CZS by gestational week of infection and (2) describe the full spectrum of CZS including neurodevelopmental milestones in the first three years of life, and (3) evaluate the social impact on families
Objective 2: To define the spectrum of neurological diseases associated with ZIKV infection, in the central nervous system and peripheral nervous system
Objective 3: To investigate the extent of and factors associated with sexual transmission of Zika in humans and mice models.
Objective 4: To discover and characterize the mechanistic pathways of ZIKV infection in the pathogenesis of CNS and PNS injury, focusing on (a) direct viral invasion, and (b) immune and autoimmune responses to viral infection.
Objective 5: To develop and evaluate diagnostic tools for the diagnosis, surveillance, and research on ZIKV.
Objective 6: To identify the major T-cell epitopes and crossreactive B-cell epitopes on ZIKV and assess the role of flavivirus antibodies in protective and pathogenic immunity following ZIKV through ADE.
Objective 7: To determine whether the contemporary ZIKV is associated with a higher viral fitness in *Aedes aegypti* mosquitoes and more severe neurological disease and testicular involvement in mice compared with historical ZIKV.
Objective 8: To assess the seroprevalence in individuals aged 2–59 years in 14 different geographic locations in Brazil, and to document and model regional and global geographic spread of ZIKV infections.
**Beyond ZIKA**:
Objective 9: To develop mathematical models to inform public health policies on how best to achieve vector control
Objective 10: To develop novel, wash-in detergent formulations and long-lasting plastic technologies containing repellents for the treatment of clothing and other wearable repellent technologies for the protection of pregnant women and nonpregnant individuals against Zika and other Aedes-transmitted diseases
Objective 11: To establish a Latin American and Caribbean network together with the other two EU-funded Zika consortia for EID preparedness to support a rapid and coherent research response to the ZIKV outbreak in the short term, and to other vector-borne and emerging infectious disease outbreaks in Latin America in the long term.
Objective 12: To develop social messaging for women of reproductive age.

## Paper context

The causal association between maternal Zika virus infection and birth defect has been established, but many other research gaps exist.

We will further define CZS, delineate neurological complications associated with Zika, investigate the role of sexual transmission, determinants of severe disease, and viral fitness, conduct burden of disease studies, and model outbreak responses. We will also build up research capacity in the region.
